# Knowledge, Attitudes, and Practices of Rural Communities Regarding Antimicrobial Resistance and Climate Change in Adadle District, Somali Region, Ethiopia: A Mixed-Methods Study

**DOI:** 10.3390/antibiotics13040292

**Published:** 2024-03-22

**Authors:** Abdifatah Muktar Muhummed, Ashenafi Alemu, Yahya Osman Maidane, Rea Tschopp, Jan Hattendorf, Pascale Vonaesch, Jakob Zinsstag, Guéladio Cissé

**Affiliations:** 1 Swiss Tropical and Public Health Institute, Kreuzstrasse 2, 4123 Allschwil, Switzerlandgueladio.cisse@swisstph.ch (G.C.); 2 Faculty of Science, University of Basel, Petersplatz 1, 4003 Basel, Switzerland; 3 Institute of Health Science, Jigjiga University, Jigjiga P.O. Box 1020, Ethiopia; 4 Armauer Hansen Research Institute, Addis Ababa P.O. Box 1005, Ethiopia; 5 Department of Fundamental Microbiology, University of Lausanne, UNIL-Sorge, 1015 Lausanne, Switzerland

**Keywords:** antimicrobial resistance, climate change, knowledge, attitude, practice, community, Ethiopia

## Abstract

There is an urgent need for interventions in addressing the rapid and disproportionate impact of antimicrobial resistance (AMR) and climate change (CC) on low- and middle-income countries. Within this context, it is important to understand indigenous knowledge in rural communities, which are highly affected. This study examined knowledge, attitude, and practices (KAP) regarding AMR and CC in the Adadle district, Somali region, Ethiopia, utilizing mixed methods, including 362 surveys and 12 focus group discussions among rural communities. Findings showed that 39% and 63% of participants were familiar with AMR and CC, respectively. Of those surveyed, 57% attributed AMR to inappropriate antimicrobial use in animals and humans, while CC was often associated with Allah/God. Multivariable analysis indicated that males exhibited superior knowledge and a positive attitude towards AMR and CC. Additionally, individuals aged 26–35 and 36–45 years showed heightened awareness of AMR and CC, respectively. Moreover, participants who were government employees, pastoralists, and business owners showed better knowledge on CC compared to family caretaker. Religious education and households with more than six members were linked to lower AMR knowledge. This study underlines a greater awareness of CC than AMR and highlights gender-based disparities, recommending integrated educational AMR programs targeting different demographics through a One Health lens, actively involving females, and incorporating local beliefs and practices.

## 1. Introduction

Globally, antimicrobial resistance (AMR) and climate change (CC) pose serious threats to public health systems and economics [1]. It is projected that 10 million lives will be lost by 2050 due to AMR, with an additional annual toll of 250,000 lives between 2030 and 2050 due to CC [2,3], and specifically its effects on malnutrition, malaria, diarrheal diseases and heat-stress [3]. AMR and CC share the commonality of having unpredictable consequences and necessitating urgent measures for control and mitigation [4]. Furthermore, there is a profound interconnection between them, with CC exacerbating and amplifying the issues associated with AMR [5].

Climate change, driven by greenhouse gas emissions from fossil fuels and human activity, directly and indirectly impacts human health [6]. Higher temperatures lead to heat-related mortality and create conditions favorable for the spread of microbes, including those with resistance genes, contributing to the rise of AMR [5,7]. CC projections anticipate an increase in both floods and droughts, which have indirect implications for human health [8,9]. In fact, population displacement due to these climatic shifts elevates the potential for zoonotic disease transmission as individuals come into closer contact with animals [9]. Furthermore, water scarcity from migration may result in shared water sources, leading to inadequate sanitation and hygiene practices, consequently heightening the risk of waterborne diseases like diarrhea [10]. These multifaceted challenges, combined with food shortages during migration, can contribute to malnutrition [11,12]. As infectious diseases become more prevalent under such circumstances, it leads to the heightened utilization of antibiotics, a well-established precursor of AMR [1]. Therefore, an increase in infectious diseases, living in crowded conditions, or experiencing malnutrition, all consequences of CC, increase the risk of acquiring antimicrobial resistant pathogens [1]. Recent studies in Europe have also confirmed the association between CC, specifically increased temperature, and AMR [1,5].

Ethiopia has experienced recurring droughts and floods in recent years, leading to tragic loss of human lives and livestock [13]. These environmental changes have also triggered outbreaks of acute watery diarrhea and malnutrition, especially in the Somali region [14]. These challenges not only contribute to increased antibiotic misuse but also elevate the risk of acquiring AMR pathogens. Several studies in Ethiopia have indicated that rural communities often possess limited knowledge about AMR [15,16]. Other studies have shown that rural communities have a better understanding of CC, which can be attributed to the direct impact it has on their livelihoods [17,18,19]. Remarkably, the knowledge and perspectives of these communities regarding CC may sometimes differ from the scientific consensus.

Given the rapid and disproportionate impact of AMR and CC on low- and middle-income countries (LMICs), such as Ethiopia, there is a critical necessity for intervention. One of the key elements in controlling AMR and adapting to CC is the indigenous knowledge held by rural populations, which plays a pivotal role in determining their adaptive capacity and resilience. Understanding how these communities perceive, engage with, and respond to these challenges is instrumental in crafting effective interventions and policies. This, in turn, paves the way for comprehensive strategies to mitigate the adverse impacts of AMR and CC.

To achieve this goal, we employed a mixed-method approach to simultaneously assess the knowledge, attitude, and practice (KAP) on AMR and CC among rural communities in the Somali region of Ethiopia.

## 2. Results

### 2.1. Quantitative Results

The characteristics of the participants in the survey are summarized in Table 1. The settlements were evenly represented with roughly half of the participants being pastoralist (50.6%) and half agro-pastoralist (49.4%).

### 2.2. Knowledge and Attitude on Antimicrobials and AMR

Most of the study population (95.9%) correctly agreed with the definition of antibiotics. However, less than half of the study participants (10–42%) accurately stated the indications for antibiotic use in common illnesses. Most of them reported that antibiotics can be used to treat watery diarrhea (89.5%), fever (71%), common cold (68.2%), and viral infections (57.7%). Moreover, most of the participants perceived that using antibiotics as injection (85.9%), costly antibiotics (92.5%) or using multiple antibiotics (85.1%) are the most effective ways to treat infections.

Among the participants, 39% had heard of AMR, with the primary sources of information being health professionals (75.8%) and radio (63.8%). Regarding AMR transmission, it was observed that more than half of the participants reported that AMR could not be transmitted between humans (74.3%), between animals and humans (54.7%), and between the environment and humans (63.3%). Notably, only 4% had heard of the antimicrobial stewardship program. The summarized results are available in Table S1.

**Figure 1:**
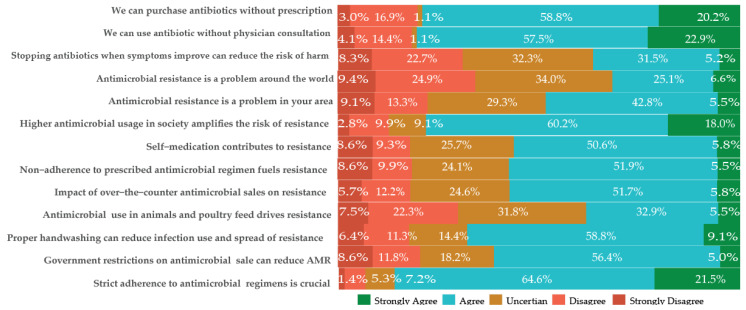
Attitude on antimicrobial use and resistance (AMR) in Adadle district, Somali region, Ethiopia.

In terms of AMR attitudes, less than half of the participants agreed that AMR is a problem locally and globally (Figure 1). Nearly 57% of the participants agreed that self-medication practices, non-adherence, and over-the-counter antimicrobial sales contribute to AMR. Additionally, 36.7% associated AMR with high antimicrobial usage in society, and 38.4% implicated animals. The measures agreed upon by the majority to reduce AMR include adherence (86.1%), proper handwashing (67.9%), and government restrictions on antimicrobial sales (61.4%). The results are summarized in Figure 1.

### 2.3. Practice on Antimicrobial Use

Among the study participants, 52.4% had used antibiotics in the past month, 17.1% in the past six months, 10.5% in the past year, and 6.3% more than a year before this study commenced. The most common diseases for which antibiotics were reported to have been used included upper-respiratory infection (75.5%), urinary tract infection (UTI, 72.7%), diarrhea (71.5%), malaria (66.6%), common cold (52.5%), and acute febrile illness (47.5%), while tuberculosis (TB, 7.2%) was the least frequently associated with reported antibiotic use. Amoxicillin (79%), ampicillin (36.5%), tetracycline (22.9%), cloxacillin (21.2%), metronidazole (19%), cotrimoxazole (11.6%), and ciprofloxacin, azithromycin, and amoxicillin with clavulanic acid (8%) constituted the antimicrobials most frequently identified by the participants as their commonly used antibiotics, as determined by their appearance (summarized in Figure S1). These antibiotics were mostly purchased from the drug store (69.3%).

**Figure 2:**
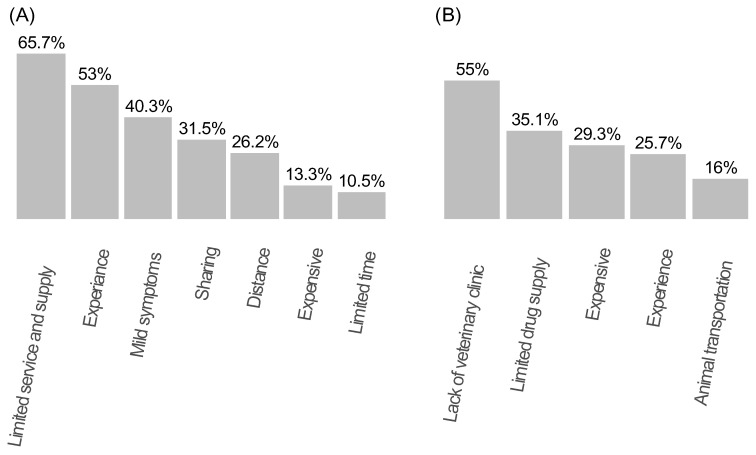
Participants’ reported reasons for not seeking health professionals (**A**) and veterinary professionals (**B**) for their livestock.

Most participants (67.4%) did not have a prescription from an authorized healthcare professional when purchasing antibiotics. The main reasons for self-medication practice included limited access to healthcare services and medication supply (65.7%), purchasing the same antibiotic when their symptoms closely resembled those of a previous illness (53%), the presence of mild symptoms (40.3%), willingness to share antibiotics with family members who exhibited similar symptoms (31%), geographical distance to healthcare facilities (26.2%), costs of antibiotics (13.3%), and limited time (10.5%) (Figure 2A). Moreover, most of the study population (62.7%) reported discontinuing medication once their symptoms had resolved.

A total of 306 participants (85%) possessed animals. The most frequently reported diseases among these animals were pasteurellosis (47.8%), diarrhea (45.6%), sheep and goat pox (43.1%), tick intoxication (22.1%), pneumonia (15.2%), anthrax (13.3%), black leg (9.1%), trypanosomiasis (8.3%), rabies (4.7%), and mastitis (3.9%). The three most used medications for these animal diseases, as cited by the participants, were oxytetracycline (83.1%), albendazole (60.8%), and procaine-streptomycin (32.6%) (summarized in Figure S1). Notably, most of the study participants (87.7%) purchased these medications without consulting veterinarians or community animal health workers (CAWS). The main reason (55%) for not visiting veterinary clinics was absence of veterinary services in their village. Participants cited several other reasons for not seeking professional veterinary care, including resource constraints (35.1%), medication affordability (29.9%), their own experience in treating and selecting medications for their livestock (25.7%), and animal transportation (16%). Results are summarized in Figure 2B.

### 2.4. Knowledge and Attitude on Climate Change

**Figure 3:**
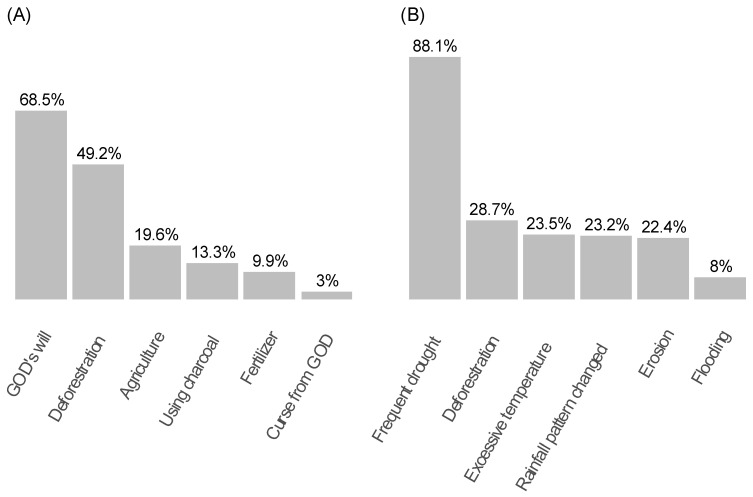
Participants’ reported causes of climate change (CC) (**A**) and emerging challenges (**B**) in the past decade.

In the community, most of the participants (63.5%) had heard of CC. Most participants (68.5%) attributed climate change to God’s will, while half of the community linked it to deforestation. Conversely, frequent droughts were the most frequently reported CC challenges (88.1%), followed by deforestation (28.7%), excessive temperatures (23.5%), changes in rainfall patterns (23.3%), and erosion (22.4%). Flooding was the least frequently reported challenge, with only 8% of participants mentioning it (Figure 3).

Among the study population, 72.4% perceived an increase in disease trends over the past five to ten years, while only 12% reported no change. The diseases reported during this period included upper respiratory infections (84.8%), diarrhea (80.1%), UTIs (76.8%), malaria (71.3%), pneumonia (67.1%), and malnutrition (53%). Less than half of the participants reported dermatological problems (22.7%), cardiovascular disease (18%), asthma (11.6%), and TB (6.6%) (Figure S2). In addition to the diseases, nearly all participants (94.5%) revealed that, due to CC, particularly drought, they were compelled to leave their residences in search of water and grazing areas for their livestock. Additionally, the other problems they faced were shortage of food (33.9%), loss of livestock (33.4%), water scarcity (30.4%), difficulty in getting access to clean water (28.4%) and health problems (26.2.%). The summarized results are available in Table S2.

Only 120 participants (33.1%) acknowledged the existence of a relationship between CC and AMR, with 21.2% perceiving that CC plays a role in the rise of AMR. Most of the community (71.8%) believed that there is a need for awareness and knowledge regarding the relationship between the two topics. Encouragingly, a substantial proportion (69.6%) of respondents expressed a keen interest in receiving updates and information pertaining to the mitigation strategies concerning both CC and AMR. Notably, a significant majority (77.3%) of these individuals favored obtaining such updates through media channels such as radio and television, while a smaller yet noteworthy portion (15.7%) indicated a preference for social media platforms, as detailed in Table S2.

### 2.5. Knowledge, Attitudes, and Practices Scores of Study Participants in Different Settings

The mean scores for K, A and P regarding CC among agro-pastoralists were 55, 60, and 20, respectively. In contrast, for AMR, the scores were 50, 56, and 40. When assessing overall KAP scores for climate change among pastoralists, we found the averages to be 51.8, 55, and 23 for K, A, and P, respectively. Comparatively, for AMR, the scores were 47, 55, and 42. Based on the mean score cut-off points, 52 and 40 of the participants demonstrated limited knowledge regarding AMR and CC, respectively. The result is summarized in Table S9.

### 2.6. Mean Knowledge and Attitude Scores of Participants Regarding AMR and CC across Demographics

The mean knowledge score for AMR and CC was higher among males than females (70 vs. 47 for CC and 63 vs. 60 for AMR). In general, older participants had highest knowledge scores. In the case of CC, participants aged 36–45 years, 25–35 years, >46 years and <25 years demonstrated a mean knowledge score of 59.1, 57.1, 50.1, and 41.6, respectively. Conversely, for AMR, the mean knowledge scores differed slightly by age group: 26–35 years (62.4), 36–45 years (61.3), and participants aged >46 and <25 years showed similar means (59). Participants with a college education or higher, those with religious education, illiterate individuals, and those with primary school showed mean scores of 73, 56, 53, and 50, respectively, in CC. In AMR, the highest mean knowledge score was shown by participants with a college and above education level (66), followed by those with religious education (61), primary school (61) and illiterate (59). In terms of marital status, the mean knowledge scores for CC were 57, 54, and 38 for widowed, married, and single individuals, respectively. For AMR, married and widowed participants showed similar mean scores (61 and 61), while singles scored the lowest mean score (58). Families composed of five or fewer members exhibited a better mean knowledge score in both CC (56) and AMR (62) compared to families with more than six members, where both groups showed similar mean scores (60). Furthermore, mean knowledge scores for both CC and AMR were consistent regardless of whether the family had lived in the area for less or more than ten years.

Regarding attitude, males, exhibited an elevated mean attitude score in CC and a slightly higher mean attitude score in AMR compared to females (66 vs. 54 of CC and 57 vs. 55 of AMR). Participants aged 35–46 and >46 years showed a higher mean attitude score, followed by those aged 26–35 years, while those <25 years registered the lowest mean score in CC. On the other hand, in AMR, almost all age groups registered similar means, between 55 and 56 (Table S1). Those with religious teaching and those with an educational level of college and above demonstrated the highest mean attitude scores of 67 and 65, respectively, whereas those with primary school education and illiterate participants showed equal mean attitude scores in CC. In AMR, only those with religious teaching showed a marginally higher mean score (58), while the rest of the educational levels showed similar mean scores (55). The detailed results are presented in Table S3 and S4.

### 2.7. Comparing the Mean Knowledge and Attitude Scores of AMR and CC

**Figure 4:**
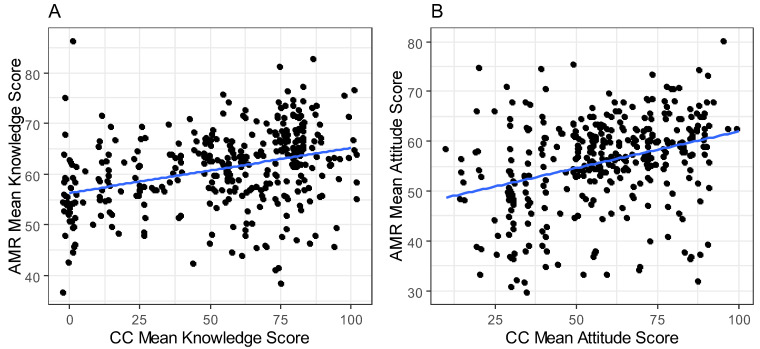
Comparison of participants’ AMR mean knowledge score and CC mean knowledge score (**A**), and participants’ AMR mean attitude score and CC mean attitude score (**B**) in Adadle district, Somali region, Ethiopia.

Figure 4 illustrates the relationship between the mean knowledge scores of AMR and CC. In Figure 4A, the scatter plot depicts a notable positive trend wherein an increase in the mean knowledge score of AMR corresponds with a concurrent increase in the mean knowledge score of CC. Similarly, Figure 4B portrays a similar positive correlation, wherein higher mean attitude scores of AMR align with elevated mean attitude scores of CC.

**Figure 5:**
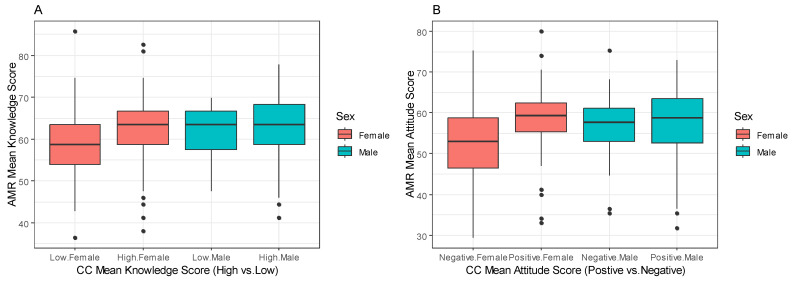
Comparing participants AMR mean knowledge score and high/low CC knowledge (**A**), and participants AMR Mean attitude score and positive/negative CC attitude (**B**) across sex in Adadle district, Somali region, Ethiopia.

In terms of sex, females who demonstrated a strong comprehension of CC showed a notably high median score in the “AMR Mean Knowledge Score”. Conversely, when their understanding of CC was limited, their median score in the “AMR Mean Knowledge Score” was correspondingly low. In contrast, for males, their median “AMR Mean Knowledge Score” remained relatively constant regardless of their level of comprehension of CC (Figure 5A). Furthermore, both females and males displayed high median scores in the “Mean AMR attitude score” when they expressed a positive attitude towards CC. Conversely, when participants exhibited a negative attitude towards CC, the median “AMR Attitude Mean Score” was low for both genders (Figure 5B).

### 2.8. Factors Associated with Knowledge and Attitudes (Multivariable Analysis)

In the multivariable analysis, we identified significant associations between specific demographic factors and knowledge levels and attitudes towards AMR and CC. Notably, being male was associated with better knowledge levels, with odds ratios (OR) of 5.48 (CI: 2.4–12.5) for AMR. Participants aged 26–35 years showed twice the odds of possessing better knowledge on AMR (OR: 2.39; CI: 1.17–4.89). Likewise, individuals in the 36–45 age group demonstrated similar odds of having twice the knowledge on CC (OR: 2.3; CI: 1.04–5.11), compared to those aged 18–25 years old. Moreover, participants whose occupations were government employees, pastoralists, and business owners, respectively, showed better knowledge on CC, with odds of (OR: 6.45; CI: 1.48–28), (OR: 5.18; CI: 2.33–11.5), and (OR: 3.5; CI: 1.5–7.92), compared to housewives.

Additionally, participants with only religious education, compared to those who were illiterate, and those living in households with more than six people, compared to households with fewer than five people, were also associated with lower knowledge levels on AMR (OR: 0.49, CI: 0.25–0.95) and (OR: 0.56, CI: 0.32–0.97).

Regarding attitudes, males had twice the odds of positive attitude towards AMR (OR: 2.64, CI: 1.28–5.47) and three times the odds of positive attitude towards CC (OR: 3.14, CI: 1.44–6.88). Participants with a religious education had positive attitude towards AMR (OR: 1.99, CI: 1.06–3.74) and CC (OR: 4.13, CI: 2.03–8.41). Conversely, participants in pastoralist and business-related occupations showed significant association with negative attitudes towards AMR, with ORs of 0.37 (CI: 0.16–0.87) and 0.33 (CI: 0.15–0.71), respectively. Additionally, being a farmer had a negative association with CC attitudes, with an OR of 0.19 (CI: 0.06–0.62). The summarized results are reported in Tables S5–S8.

### 2.9. Qualitative Results

In the focus group discussion (FGD), most of the Kebeles had an equal number of participants, of which 50 (53.1%) were male and 46 (47.9%) were female. Most participants (77) were illiterate (80.2%). In total, 11 (11.4%) went to college, 6 (6.3%) reached high school, and 2 (2.1%) went to primary school. Participants’ characteristics are presented in Table 2.

### 2.10. Knowledge on Antimicrobials and Antimicrobial Use

Most participants of the FGD were able to associatiote antimicrobials based on the color of the capsules, particularly red and black, which are commonly used for ampicillin capsules known as “qoormadoobe” in the Somali language. Moreover, some participants demonstrated the ability to identify specific antimicrobials by their names, such as amoxicillin and ampicillin.

*Aaah’ whenever we go to the health facility or pharmacy, they always say it is an infection and give us Amoxicillin, which I believe is not as effective as Ampicillin (qoormdoobe, or black neck). The two most common antibiotics that we use are amoxicillin and ampicillin (qoormadoobe, or black neck)*

[CM: Woman: Age: 42 years]

*They are drugs that can treat any diseases or alleviate pain, and without them, I believe life would have been very difficult for us.*

[CM: Man: Age: 54 years]

### 2.11. Knowledge on AMR

Most participants had a limited knowledge of AMR, only few participants were able to share their ideas and draw from personal experiences with antibiotic use after being prompted and provided with a simple explanation of antimicrobial resistance.

*Yeah, we often hear that, if we do not take the medication correctly or misuse it, our bodies can adapt to it, which means it may not work in the future.*

[CM: Man: Age: 52 years]

Regarding gender, women were reported to be more susceptible to diseases and tend to rely on antimicrobial drugs more than men. They often expressed dissatisfaction with the effectiveness of current medications, attributing it to the lack of diagnostic facilities and a mismatch between the disease and the prescribed treatment. Furthermore, there is a prevailing belief among some women that older medications are more effective than those currently available. In fact, some have gone as far as to claim that “both people and medications from the past were of better quality”.

*I have heard several times that people complain about antibiotics like amoxicillin not working. I have experienced this myself—I took another antibiotic, and it did not work either. However, I never considered it might be due to resistance; I simply thought maybe the disease and the medicine didn’t match. There are many such cases.*

[CM: Woman: Age: 49 years]

Most participants had poor knowledge regarding the source and spread of resistant pathogens between humans, animals, and the environment. Some participants knew resistant pathogens can spread from humans to humans, such as drug-resistant TB, but not to/from animals or the environment.

*Yes, it can be transmitted, for example, if one person is infected with TB, he/she can transmit it to the other family members who live with them or share food or are in close proximity to them. We used to take them outside the house and keep them away from the family. Because we know that, they can transmit to the other family member. If he gets close to the family member.*

[CM: Female: Age: 49 years]

Overall, community participants had never heard about the AMR stewardship program.

### 2.12. Attitude and Practices on Antimicrobial Use

Most participants found it difficult to adhere to the prescribed antibiotic regimen. The most common habit was not finishing the prescribed antimicrobials, either because of a deliberate decision to stop taking the medication after feeling better or forgetting it because of workload. Severe illness was reported as one of the main drivers of adherence.

The participants agreed upon sharing antimicrobials with neighbors or family members, particularly if they have similar symptoms. A small number of community participants had differing opinions on not using leftover antimicrobials. Overall, participants reported the disposal of leftover/unused medicine as part of household waste.

*Yes, we ask and share medicine within ourselves. For example, if we share the same signs and symptoms with a family member or neighbor, like coughing, I will share the antibiotic with them. Sometimes, I keep it for future use in case somebody gets sick.*

[CM: Woman: Age: 51 years]

According to most participants, self-medication is extensively practiced in the region. Most participants stated that it was easy to purchase antibiotics without prescription from pharmacies or drug stores. This is especially true for symptoms such as coughing or mild diarrhea, which are often considered minor illnesses. Most participants stated that they also self-medicated in cases in which the symptoms were the same as those of a prior disease.

Other common characteristics associated with self-medication include insufficient drug supply at the health facility, time, cutting the costs of doctor consultation, education, medical staff behavior, fear of being diagnosed with another disease, and patient behavior.

*I would like to go to the health facility, but I have to take care of the children, the house, and other family activities. Therefore, it is easier and quicker to get the drugs from the pharmacy instead of going to the hospital, which takes all morning.*

[CM: Female: Age: 45 years].

*We prefer the health center because the medications there are cheaper and of good quality compared to outside pharmacies. However, insufficient drug supply, inappropriate diagnosis, lack of laboratory services, and a limited number of health professionals often compel us to go to the pharmacy instead. This is because pharmacies may have a better drug supply than the health center. Additionally, the health professionals who work in the health center often own the pharmacy. The services they provide are essentially the same since both lack basic investigation.*

[CM: Female: Age: 37 years].

### 2.13. Livestock Antimicrobial Use and Practice

The community reported that the most observed livestock diseases were runny nose, diarrhea, pasteurellosis, sheep and goat pox, and pneumonia. It was noted that most of the community used antibiotics such as oxytetracycline and penstrep without seeking advice from community animal health workers.

*One of the challenges we face is the limited availability of medication for livestock in our area. Throughout the year, we receive only a small number of vials of tetracycline and penstrep. Tetracycline is the only drug accessible for treating livestock, and we utilize it whenever it is available or when there are remaining doses. In situations involving severe conditions, we consult animal health workers. However, their capacity to provide comprehensive assistance is constrained by the limited availability of animal health services. Regardless of whether we consult with professionals or not, the situation remains unchanged. Consequently, we continue to rely on our experiences to address the health needs of our livestock.*

[CM: Community animal health worker: Age: 45 years].

### 2.14. Climate Change and Antimicrobial Resistance

The terms “CC” and “weather” were widely misunderstood by respondents. After a brief explanation of CC, most of the community acknowledged it and recounted their experiences with recurring droughts.

*The weather is getting worse year after year. Well… let me tell you. In the past eight to ten years, we have been tussling with severe droughts. For ten years now, we used to name the droughts because they occurred one at a time for extended periods. However, in the last eight years, droughts have been happening consecutively. Due to their recurrence, we no longer give them names.*

[CM: Female: Age: 41 years].

*Over the past eight years, we have lost most of our livestock to drought rather than diseases. To save the remaining livestock, most of the community migrated from the Kebele in search of water and food. Whenever we face severe drought, we usually migrate to find water and food, but during these migrations, we lose some of our livestock due to hunger and diseases.*

[CM: Male: Age: 54 years].

*Well … we do not have the technology or materials to measure the weather, only God knows the change of the weather.*

[CM: Female: Age: 43 years].

Participants commented on how the disease pattern has changed in their families or communities over the last five years. Most people in the community said that there was a link between droughts and infectious illnesses such as diarrhea, upper respiratory infections, and malnutrition.

## 3. Discussion

AMR and CC are important, current issues that affect the entire world population, and will increasingly do so in the future [5]. Multiple aspects of biological, economical, socio-cultural and political nature must be taken into account when studying and addressing them [1]. Furthermore, their interconnection and the effect the one has on the other must also be considered [1]. In fact, CC may have an impact on AMR, as drought and flooding—and the living conditions resulting from them—may increase infection rates, leading to an increase in misuse of antimicrobials [20], which is the main driver of AMR [21]. Vulnerable, rural populations in the Global South are likely highly impacted by both AMR and CC [6,21], but often lack knowledge or means to understand and address them. Therefore, in this study we assessed the knowledge, attitudes and practices of rural communities in Adadle district, Ethiopia, regarding AMR and CC, with the aim of increasing awareness and laying the foundation to support these communities in controlling AMR and living with CC in the future.

In general, respondents demonstrated a lack of knowledge concerning both AMR (52.5%) and CC (40%). Our findings are in alignment with recent systematic reviews conducted in Ethiopia [22] and communities in Dessie [15], which reflect the same trends for AMR awareness. Moreover, other studies conducted in different rural communities in Ethiopia, Kenya, and South Africa similarly reported a commendable level of knowledge regarding CC [23,24]. This confirms our predictions of rural communities lacking knowledge about these important issues, and highlights the need for interventions in this regard. Notably, in both qualitative and quantitative analyses, participants were found to be more knowledgeable on CC than on AMR, suggesting that the direct impact of CC on rural livelihoods may contribute to their heightened sensitivity and awareness to it.

Our results, combined with other results from Ethiopia and other East African countries [15,25,26,27,28], link the cause of AMR to three main factors: (1) self-medication practice, (2) over-the-counter sale of antibiotics, and (3) inappropriate use of antibiotics in humans and animals. All of these were found to be highly practiced in the community and livestock in our study, and could be attributed to their precarious socio-economic conditions, limited accessibility to adequate infrastructure and lack of knowledge about correct antimicrobial use.

Indeed, the primary drivers of self-medication practices among humans in our study and other studies encompass limited access to healthcare services and supplies, prior personal experiences, and the severity of the ailment [29,30]; while for livestock, the major factors include the absence of veterinary clinics, limited drug availability, cost considerations, and prior experience. Additionally, the financial burden of medical visits play a significant role in promoting self-medication [31]. This practice is further facilitated by the accessibility of over-the-counter antibiotics, which are most often sold without a prescription. This sheds light on the challenging implementation of government regulations on antimicrobial use and sale in rural communities.

These observations emphasize the necessity for of developing integrated educational and stewardship programs on antimicrobial use in both humans and animals and of strengthening regulatory measures regarding over-the-counter sale of antimicrobials. Additionally, there is an overall need for improving healthcare infrastructure and accessibility, making services more affordable, and expanding veterinary clinics to ensure proper animal care.

The third main driver of AMR, namely the inappropriate use of antimicrobials, has been previously attributed to stopping treatment after signs and symptoms faded or simply forgetting to take them [21,32]. Unused antimicrobials might be given to a family member or neighbor exhibiting similar signs and symptoms, stored for future use, or discarded as household waste [33,34,35]. A recent review in an East African pastoralist setting reported that misuse of antimicrobials in humans and animals significantly contributes to antimicrobial resistance (AMR) in these regions [36]. These findings align with our qualitative and quantitative results. This illustrates how essential counseling by health professionals is when prescribing medicines, particularly antimicrobial drugs, to ensure that patients understand the importance of taking the medication as directed and the risks of not doing so. This can improve health outcomes and reduce the risk of developing antibiotic resistance.

Regarding CC, most our respondents acknowledged CC, with a particular emphasis on the frequent droughts, deforestation, excessive heat, and a reduced predictability of rainfall. However, these communities often attributed these changes to Allah or God [37,38,39,40,41], as most of our study participants also reported. This perspective is deeply entrenched in cultural and religious beliefs, often transmitted across generations. While these beliefs offer solace and explanations for the inexplicable, they can impede the comprehension of CC as a scientific phenomenon driven by human activities, including the combustion of fossil fuels, deforestation, and industrial processes [42]. Addressing this aspect requires a delicate, transdisciplinary approach that respects local beliefs while also introducing scientific knowledge. For instance, the Bidirectional Emic-Etic tool (BEE) has been employed to bridge the gap between traditional beliefs and scientific understanding concerning intercultural differences [43]. This approach has proven effective in addressing societal challenges related to environmental sustainability and is recommended for addressing issues related to CC [44,45].

Additionally, deforestation was another reason that our participants attributed to CC, which aligns with studies in Nigeria and Bangladesh [46,47]. This highlights the pressing need for education and awareness campaigns in rural communities, which heavily rely on forests for their livelihoods and energy. These campaigns should not only emphasize the environmental consequences of deforestation but also promote practical alternatives, like stoves powered by solar energy [48].

Despite the critical interplay between CC and AMR, our study revealed a stark lack of awareness among the majority of respondents regarding this crucial connection. Less than half of those surveyed acknowledged the correlation between CC and AMR, highlighting a significant gap in understanding within the public domain. Interestingly, despite this lack of awareness, there was widespread recognition among participants of the escalating trend in disease prevalence over the past decade. This trend can be largely attributed to the escalating challenges posed by climate change, including the frequent droughts and flooding [14,49]. Among the challenges highlighted by respondents, recurrent droughts emerged as particularly profound, exacerbating issues such as food insecurity, water scarcity, and the loss of livestock [14,49]. The severity of these challenges often forces individuals to flee from their homes in search of more sustainable livelihoods [50]. This displacement frequently results in increased proximity between humans and animals, as well as overcrowding at water sources [9]. Consequently, these conditions create fertile ground for the spread of infectious pathogens within these populations [51,52]. Consistent with findings from numerous other studies, our research underscores a notable surge in infectious diseases among displaced populations, including respiratory infections, diarrhea, and vector-borne diseases [23,53]. This surge in infectious diseases is often compounded by the misuse of antibiotics, a significant contributing factor to the development of AMR [54].

When analyzing the impact of the climate crisis on the proliferation of infectious diseases and drug-resistant bacteria, the nexus between AMR and CC becomes indisputably apparent. Addressing this knowledge gap is essential for developing integrated educational programs to mitigate the intertwined challenges of climate change and antimicrobial resistance. Remarkably, rural communities have demonstrated a keen interest in acquiring knowledge and awareness pertaining to the correlation between CC and AMR, as well as strategies for their mitigation though radio/TV and social media. Leveraging innovative communication platforms such as radio, television, and mobile health applications can serve as effective channels for disseminating educational programs tailored to address these pressing issues [55]. By integrating information on climate change and antimicrobial resistance into such communication channels, educational initiatives can reach rural communities and facilitate greater understanding and adoption of mitigation measures [55].

In the multivariable analysis, it was determined that males exhibited significantly higher levels of awareness and a more positive attitude toward AMR and CC than females. Similar results were reported in Ethiopia, Tanzania, and Nigeria for AMR [15,27,56], and in Ethiopia and Bangladesh for CC [47,57]. Conversely, recent reviews have indicated that females tend to be more knowledgeable about AMR [58]. This has been attributed to the fact that females are more often exposed to antimicrobials throughout their lifetimes, which leads to greater awareness about antibiotics and AMR. This discrepancy in findings could be attributed to the cultural distinctions in the study setting, particularly in Ethiopia, where a significant gender gap exists in higher education enrollment, favoring males [15]. This enrollment discrepancy may explain the greater knowledge demonstrated by males regarding AMR and climate change compared to females, as previous research has consistently shown a positive association between higher levels of education and a better understanding of AMR and CC [16,59,60,61]. Additionally, in rural communities, male are often seen as the head of households, facilitating their involvement in meetings, training sessions, media exposure, and information sharing, which is culturally accepted to primarily occur among men [62]. This underscores the importance of an integrated educational program addressing AMR and CC, with a particular focus on involving female participants. WHO and other studies have stressed that achieving gender equity holds practical significance in tackling both AMR and CC [63,64].

Our study, along with other findings, highlights that adults generally possess a better understanding of AMR and CC compared to young adults [27,65,66]. This could be attributed to the fact that adults’ increased vulnerability to both infectious and chronic diseases might contribute to their AMR knowledge, as it could lead to greater exposure to health information from healthcare professionals, thus enhancing their understanding [15,67]. This finding suggests a potential correlation between age and knowledge proficiency, potentially attributable to accrued life experiences, prolonged exposure to informational resources, as well as increased societal recognition and leadership roles within communities, facilitating easier access to information dissemination channels of both AMR and CC, whether through governmental initiatives or specialized training programs [68].

In addition, our FGD, participants shared experiences regarding naming drought events over the last ten years. They mentioned ceasing to name them due to the increased frequency of drought occurrences. This observation also highlights how being adults has heightened their awareness of CC. This potential correlation between age, knowledge acquisition, and societal standing underscores the multifaceted nature of the dynamics shaping awareness and comprehension of critical issues such as CC. In contrary, in a study conducted in Singapore, adults exhibited illusory knowledge on CC [69]. This underlines the complex interplay of cultural, social, and contextual factors in shaping attitudes and behaviors related to CC. These findings emphasize the importance of considering local contexts and generational perceptions when planning interventions or communication strategies aimed at addressing these issues.

Participants whose occupations were government employees, pastoralists, and business owners showed better knowledge on CC compared to housewives. This discrepancy can be attributed to the fact that the government employees generally have better access to information and training opportunities related to CC provided by different stakeholders. Additionally, their level of education emerges as a significant predictor of CC awareness [70]. Regarding business owners, the observed difference in knowledge can be elucidated by the nature of their establishments, often serving as hubs for community discussions. These interactions facilitate information accessibility and contribute to a deeper understanding of CC. Additionally, the typically higher-income levels of business owners may contribute to their better understanding of CC, as observed in previous studies linking higher income with better knowledge on CC [69,71]. This finding advocates empowering women in both educational, political, and economic spheres, as they are main components for mitigating climate change [72].

In summary, our study concurrently assessed the KAP pertaining to AMR and CC. Our findings revealed that participants exhibited a greater level of familiarity with CC in comparison to AMR. This discrepancy is attributed to the direct impact of CC on rural livelihoods. However, it is crucial to note that this CC knowledge did not consistently align with established scientific understanding. Furthermore, our analysis indicated a notable gender-based disparity, with males exhibiting a higher level of comprehension in both AMR and CC-related domains. Given the multidimensional nature of these issues and their intricate interplay, we propose implementing community-based educational programs or policy interventions to promote responsible antimicrobial use and environmental conservation. These interventions should target different demographic strata, including farmers and pastoralists, employing a comprehensive transdisciplinary approach. It is imperative to emphasize the active involvement of females in educational initiatives, while being sensitive to and incorporating local beliefs and practices into the pedagogical framework. Furthermore, we recommend that future research adopting a One Health approach involve a broader spectrum of expertise, including health professionals, veterinarians, environmental scientists, and social scientists.

## 4. Materials and Methods

### 4.1. Study Area

**Figure 6:**
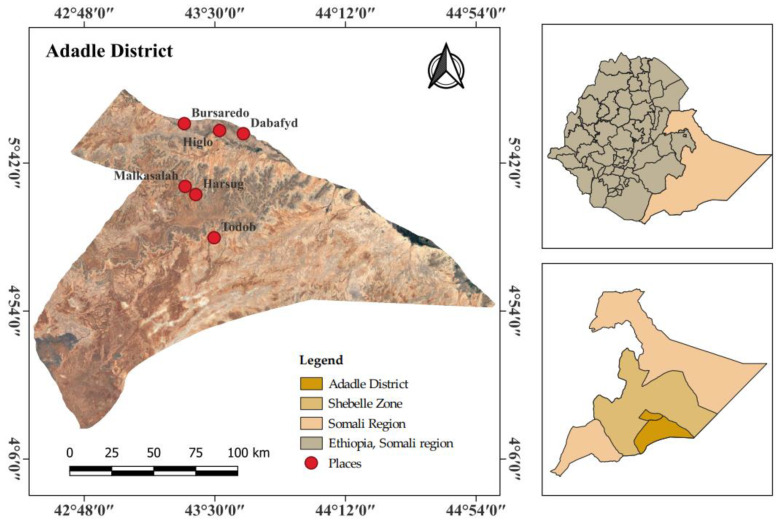
Map of the study area, Adadle district, Somali region, Ethiopia. Map created using QGIS.

This study was conducted in Adadle, a district located within the Shebele zone of the Somali Regional State in Eastern Ethiopia (Figure 6).

### 4.2. Study Desing

A mixed-methods approach was employed to assess the knowledge, attitude, and practice (KAP) of AMR and CC among pastoralist and agro-pastoralist populations.

### 4.3. Sample Size Calculation

Based on a previous study conducted in Bahardar, Ethiopia, we expected the prevalence of the knowledge level of AMR to be 42% [16] and assumed an intercluster correlation coefficient of 0.15. We calculated a sample size of 360 participants, which would be sufficient to estimate the prevalence of knowledge, with a margin of error of 10% at the 95% confidence level.

### 4.4. Data Collection Instrument

After reviewing the published literature, a semi-structured interview guide and survey questionnaire were developed [73,74,75,76]. A pilot study was conducted to ensure the clarity of the questionnaire and amendments were made accordingly. The interview guide and survey questionnaire were translated into the local language (Somali) and administered to the participants, whose responses were translated back to English [77]. Most of the questions were open-ended, with sequential prompts as needed, to enable free discussion. This encouraged the participants to elaborate and share different examples on the topic, aiding in collecting a comprehensive dataset. The interview guide for the survey and focus group discussions comprised five sections: (i) socio-demographic characteristics; (ii) knowledge of antimicrobials and antimicrobial use; (iii) KAP of AMR; (iv) knowledge of antimicrobial use in animals; (v) KAP of CC and its relation to AMR.

### 4.5. Sampling Technique and Data Collection

Out of the 13 kebele (villages) of the Adadle district, six were randomly selected. Community leaders and elders in Adadle district were engaged in the selection of households included in this study. In this survey, we conducted interviews with 362 participants in June, 2023, from three pastoralist communities (Malkasalah, Todab, Harsug) and three agro-pastoralist communities (Bursaredo, Dabafyd, Higlo). For household selection, the kebele leaders provided a list of households in the kebele. Based on this list, we generated a random number using R to select households. In cases where a selected household was not available during data collection, it was immediately substituted with the next household that was not previously selected.

Twelve focus group discussions (FGD) with community members were conducted with 8–9 participants to better understand the community KAP on AMR and CC. The selection of participants considered having an equal representation of the different social strata, such as gender, community health worker, community animal worker, community leaders, and religious leader. The moderators ensured that the participants felt free to express their views and experiences to uncover the degree of consensus or variety on the topic. Each FGD lasted from 60 to 90 min, and audio recordings and notes were taken with the participants’ consent. The interviewees continued to interview until the point of saturation [78,79]. By the tenth interview, saturation was achieved for the FGD, but two additional interviews were conducted to confirm saturation.

### 4.6. Data Analysis

R statistical software version 4.1.3 was used to perform the statistical analysis. Descriptive analysis was carried out initially for all the variables to gain an overview of the data using the gtsummary package. For the knowledge, attitude, and practice responses, a scoring scheme was employed. For binary responses, a score of “1” was assigned to correct responses, and “0” to incorrect responses. In the case of questions with multiple responses, we split each response into binary format using the grepl function in R. A score of “1” was assigned for each correct response, and “0” for incorrect responses. Similarly, for Likert scale questions, answers were assigned a range from 1 to 5 based on the selected response. A sum score above the mean was categorized as “good knowledge”, while a sum score below the mean was categorized as “poor knowledge”. Likewise, a sum score greater than the mean assigned a “favorable attitude”, and a sum score less than the mean assigned an “unfavorable attitude”.

Multivariate analysis was performed to determine the association between binary outcome (good vs. poor knowledge and favorable vs. unfavorable attitude) and independent variables using a logistic regression. The logistic regression analysis was conducted using the ‘glmer’ package. Initially, an inclusive model was created, encompassing all variables (age, sex, education, marital status, occupation, number of people per household, and years lived in the area), based on prior literature, while adjusting the cluster. In a stepwise manner, we iteratively improved the model by removing variables that did not significantly contribute (*p* > 0.2) while considering their impact on the overall model fitness. Variables with a *p*-value less than 0.2 were retained in the model. We used the likelihood ratio test, AIC (Akaike Information Criterion), and adjusted R-squared to assess the model’s goodness of fit. Variables with a *p*-value less than 0.05 were deemed statistically significant.

For the qualitative analysis, all interviews were audio-recorded and transcribed verbatim. The thematic analysis was performed by multiple independent analysts [80]. Before analysis, we familiarized ourselves with the data to have an overview of all collected data. Independent researchers (A. Muhummed and Y. Osman) coded the documents simultaneously using inductive process with Atlas.ti (version 8.4). Each researcher shared and discussed the meaning of the codes. Subsequently, the codes underwent a cross-check for intercoder reliability, calculated using a simple percent agreement method [81], resulting in a 92% agreement. In instances of notable disagreement, an additional coder with expertise in qualitative analysis was consulted to address and resolve the outstanding discrepancies (A. Kaiser-Grolimund). The generated codes were grouped and converted into themes. The researchers (A. Muhummed, A. Kaiser-Grolimund, and Y. Osman) then reviewed these themes to ensure they accurately reflected the meaning and nuances of the coded data [82]. Following the review, a consensus was reached on naming the themes, along with their corresponding codes and supporting evidence from the dataset [82].

## Figures and Tables

**Table 1:** Demographic characteristic of participants in Adadle district, Somali region, Ethiopia.

Variables	N = 362 (%)
Settlement	
Pastoralist	183 (50.6%)
Agro-pastoralist	179 (49.4%)
Sex	
Female	257 (71.0%)
Male	105 (29.0%)
Age group (in years)	
<30	141 (39.0%)
31–40	112 (30.9%)
>40	109 (30.1%)
Marital status	
Single	25 (6.9%)
Married	327 (90.3%)
Divorced	3 (0.8%)
Widowed	7 (1.9%)
Educational status	
Illiterate	235 (64.9%)
Primary school	58 (16.0%)
Religious learning	63 (17.4%)
College and above	6 (1.7%)
Occupation	
Housewife	212 (58.6%)
Government employee	19 (5.2%)
Pastoralist	56 (15.5%)
Farmer	23 (6.4%)
Business	52 (14.4%)
Numbers of person per household	
<5	129 (35.6%)
6–8	121 (33.4%)
>8	112 (30.9%)
How long they have lived in the area	
<10 years	153 (42.3%)
>10 years	209 (57.7%)

**Table 2:** Background characteristics of the community members taking part in the Focus group discussion (FGD) (N = 96) in Adadle district, Somali region, Ethiopia.

Variable	N = 96 (%)
Sex	
Female	46 (47.9%)
Male	50 (53.1%)
Age	
18–25	22 (22.9%)
26–35	35 (36.5%)
>36	39 (40.6%)
Level of education	
Illiterate	77 (80.2%)
Primary school	2 (2.1%)
Secondary school	6 (6.3%)
College and above	11 (11.4%)
Occupation	
Farmer	30 (31.2%)
Housewife	22 (22.9%)
Government	15 (15.6%)
Business owner	12 (12.5%)
NGOs	17 (17.8%)
Village of residence	
Bursaredo	17 (17.7%)
Gabal	16 (16.6%)
Malkasalah	16 (16.6%)
Harsug	15 (15.6%)
Dabafayd	16 (16.6%)
Todob	17 (17.7%)

## Data Availability

On reasonable request, the corresponding author will provide the datasets used or analyzed during the current study.

## Supplementary Materials

The following supporting information can be downloaded at: https://www.mdpi.com/article/10.3390/antibiotics13040292/s1, Table S1: The knowledge of antimicrobials and antimicrobial resistance in Adadle district, Somali region, Ethiopia. Table S2: The knowledge and attitude of climate change and AMR in Adadle district, Somali region, Ethiopia. Table S3: Predictors of knowledge towards climate change in Adadle district, Somali region, Ethiopia. Table S4: Knowledge and attitude scores of the participants by socio-demographic variables regarding climate change in Adadle district, Somali region, Ethiopia. Table S5: Knowledge and attitude scores of the participants by socio-demographic variables regarding antimicrobial resistance in Adadle district, Somali region, Ethiopia. Table S6: Predictors of attitude towards climate change in Adadle district, Somali region, Ethiopia. Table S7: Predictors of knowledge towards antimicrobial and antimicrobial resistance in Adadle district, Somali region, Ethiopia. Table S8: Predictors of attitude towards antimicrobial and antimicrobial resistance at multivariable level in Adadle district, Somali region, Ethiopia. Table S9: the percentage of householders’ knowledge, attitude, and practice (KAP) scores in Adadle district, Somali region, Ethiopia. Figure S1: Participants reported common human (A) and animal diseases (C), as well as antimicrobial medications used by the participants (B) and for their livestock (D) in Adadle district, Somali region, Ethiopia. Figure S2: Participant’s reported illnesses that they have experienced over the past five to ten years.
