# Supplementary material for: Knowledge, Attitudes, and Practices of Rural Communities Regarding Antimicrobial Resistance and Climate Change in Adadle District, Somali Region, Ethiopia: A Mixed-Methods Study
Source: Antibiotics (Basel). 2024 Mar 22;13(4):292. doi: 10.3390/antibiotics13040292 (PMC11047595; doi:10.3390/antibiotics13040292)
Supplement: Supplementary file 1 [file antibiotics-13-00292-s001.zip › antibiotics-2910333-supplementary.pdf]

antibiotics

MDPI

Knowledge, Attitudes, and Practices of Rural Communities Regarding Antimicrobial Resistance and Climate Change in Adadle District, Somali Region, Ethiopia: A Mixed-Methods Study

Abdifatah Muktar Muhummed [1,2,3,*], Ashenafi Alemu [4], Yahya Osman Maidane [1,2,3], Rea Tschopp [1,2,4], Jan Hattendorf [1,2], Pascale Vonaesch [5], Jakob Zinsstag [1,2] and Guéladio Cissé [1,2]

[1] Swiss Tropical and Public Health Institute, Kreuzstrasse 2, 4123 Allschwil, Switzerland; maidane.osman@swisstph.ch (Y.O.); rea.tschopp@swisstph.ch (R.T.); jan.hattendorf@swisstph.ch (J.H.); jakob.zinsstag@swisstph.ch (J.Z.); gueladio.cisse@swisstph.ch (G.C.)
[2] Faculty of Science, University of Basel, Petersplatz 1, 4003 Basel, Switzerland
[3] Institute of Health Science, Jigjiga University, *P.O. Box 1020 Jigjiga, Ethiopia*
[4] Armauer Hansen Research Institute, P.O. Box 1005 Addis Ababa, Ethiopia; ashenafi.alemu@ahri.gov.et
[5] Department of Fundamental Microbiology, University of Lausanne, UNIL-Sorge, 1015 Lausanne, Switzerland; pascale.vonaesch@unil.ch
* Correspondence: abdifatah.muhummed@swisstph.ch

Supplementary Materials

Table S1. The knowledge of antimicrobials and antimicrobial resistance in Adadle district, Somali region, Ethiopia.

Variables	N = 362[1]
Antibiotics can treat common cold	
No	115 (31.8%)
Yes	247 (68.2%)
Antibiotics can treat diarrhea	
No	38 (10.5%)
Yes	324 (89.5%)
Antibiotics can kill virus	
No	150 (41.8%)
Yes	209 (58.2%)
Antibiotics should always be used to treat fever	
No	105 (29.0%)
Yes	257 (71.0%)
Combination antibiotics accelerate infection cure	
No	39 (10.8%)
Yes	323 (89.2%)
Injectable antibiotics are more potent than oral ones	
No	51 (14.1%)
Yes	311 (85.9%)
Costly antibiotics are powerful	
No	27 (7.5%)
Yes	335 (92.5%)
Participant has heard about AMR	
No	221 (61.0%)
Yes	141 (39.0%)
Human-to-human AMR transmission	
No	269 (74.3%)

Yes	93 (25.7%)
Human-to-animal AMR transmission is possible	
No	198 (54.7%)
Yes	164 (45.3%)
Human-to-environment AMR transmission is possible	
No	229 (63.3%)
Yes	133 (36.7%)
Participant has heard about stewardship programs	
No	345 (95.3%)
Yes	17 (4.7%)

[1]n (%),

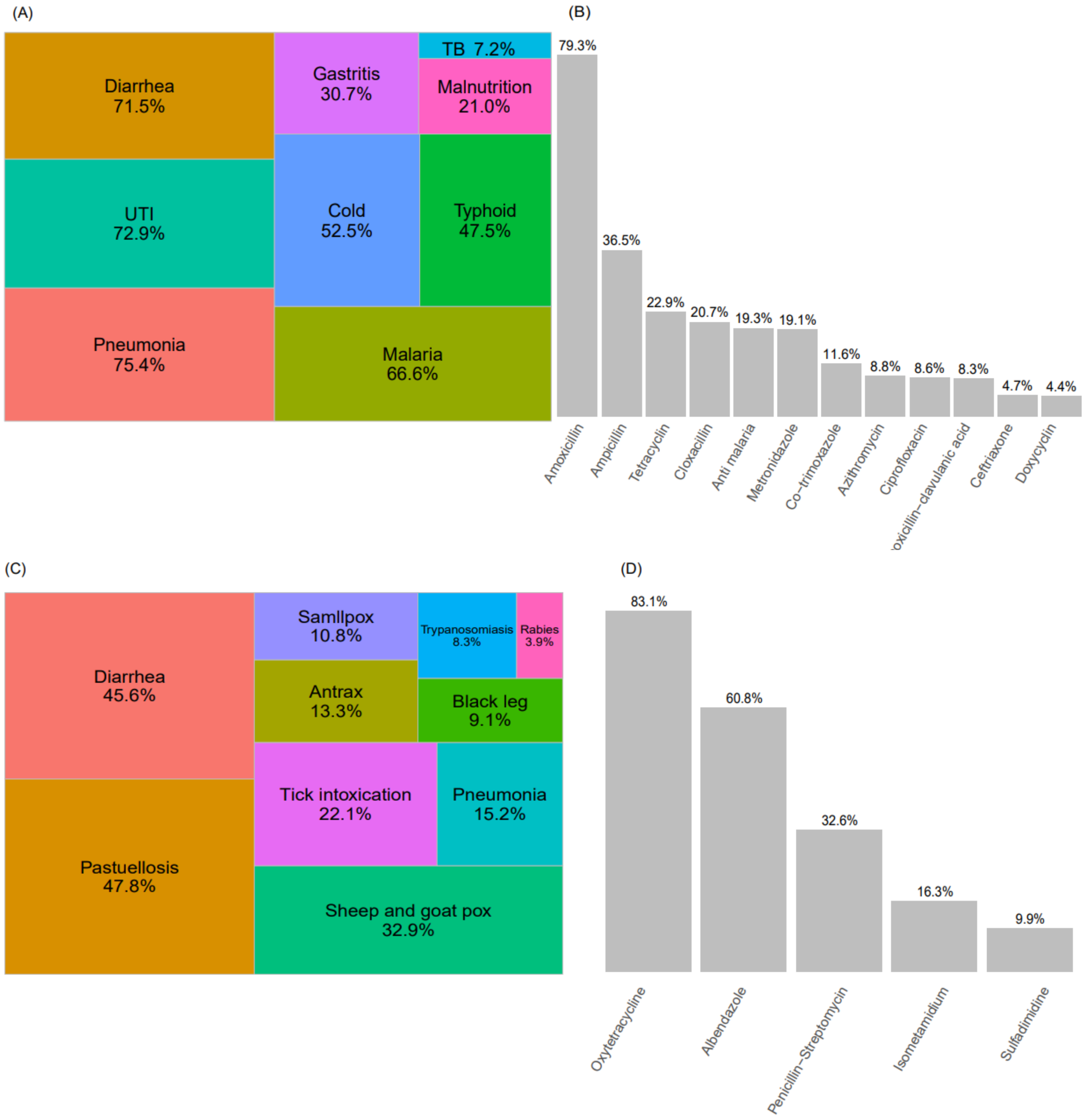

Figure S1. Participants reported common human (A) and animal diseases (C), as well as antimicrobial medications used by the participants (B) and for their livestock (D) in Adadle district, Somali region, Ethiopia.

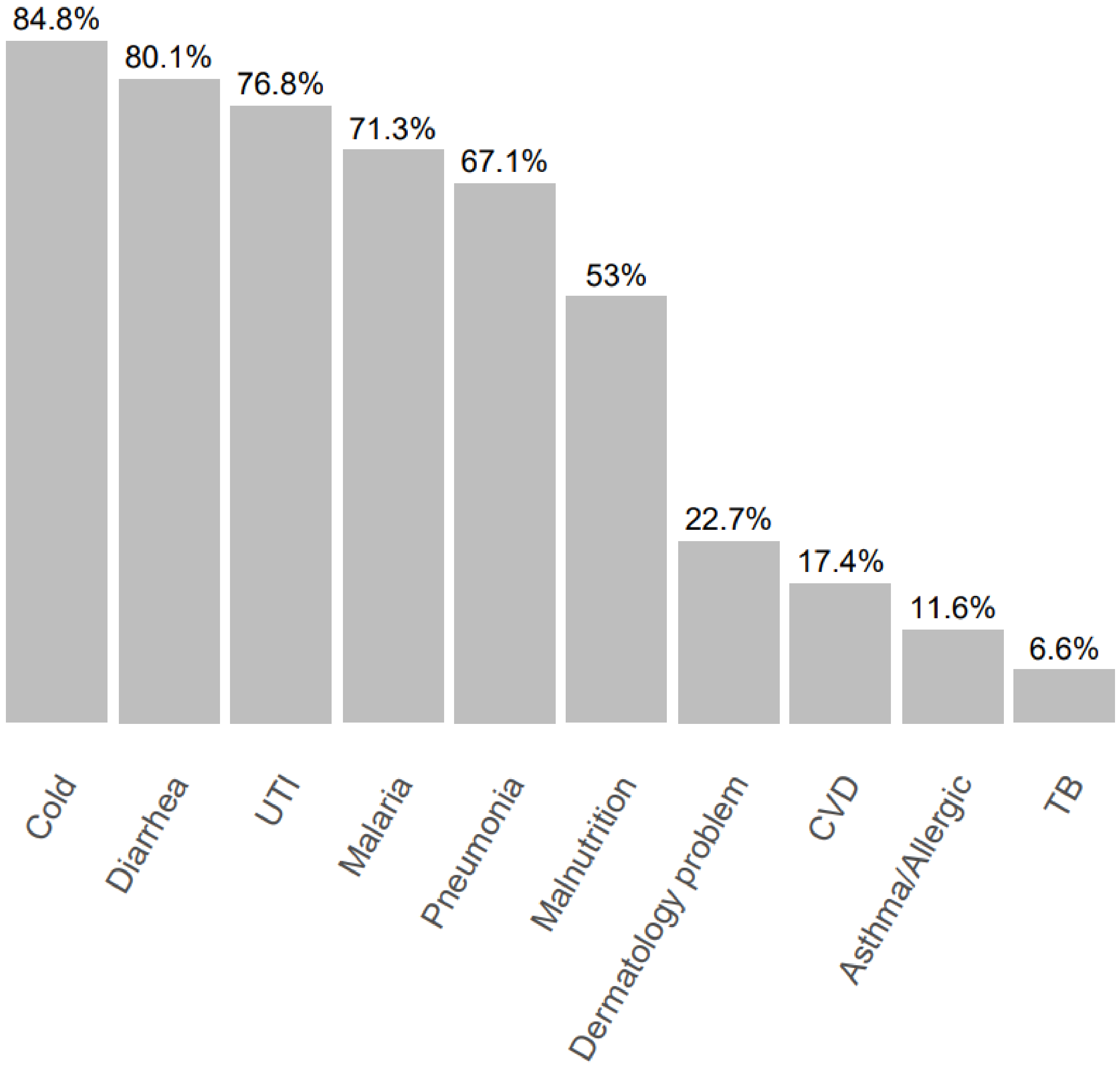

Figure S2. Participant's reported illnesses that they have experienced over the past five to ten years.

Table S2. The knowledge and attitude of climate change and AMR in Adadle district, Somali region, Ethiopia.

Variables	N = 362[1]
Participants has heard climate change	
No	132 (36.5%)
Yes	230 (63.5%)
Challenges faced due to climate change	
Forced to move out from the shelters	342 (94.5%)
Lack of food due to drought	123 (33.9%)
Loss of livestock	121 (33.4%)
Water scarcity	110 (30.4%)
Access to clean water	103 (28.4%)
Health problems	95 (26.2%)
Climate change and AMR	
Relationship between climate change and AMR	

No	242 (66.9%)
Yes	120 (33.1%)
How serious Climate change and AMR at your area?	
Not serious	71 (19.3%)
Moderately serious	131 (36.2%)
Extremely serious	36 (9.9%)
Don't know	125 (34.5%)
Should the government take responsibility for controlling the spread of AMR due to climate change?	
No	169 (46.7%)
Yes	193 (53.3%)
Need of awareness of climate change and AMR	
No	102 (28.2%)
Yes	260 (71.8%)
Desire to receive updated information on mitigation measures related to climate change and AMR	
No	110 (30.4%)
Yes	252 (69.6%)
Preferred way to receive updated information	
TV/Radio	280 (77.3%)
Social media	57 (15.7%)

[1]n (%),

Table S3. Knowledge and attitude scores of the participants by socio-demographic variables regarding climate change in Adadle district, Somali region, Ethiopia. .

Variables	**Pastoralist**		**Agro-pastoralist**		**Overall**	**Overall**
	Knowledge Mean (SD)	Attitude Mean (SD)	Knowledge Mean (SD)	Attitude Mean (SD)	Knowledge Mean (SD)	Attitude Mean (SD)
Sex						
Female	46.1 (29.3)	51.4 (19.8)	48.7 (31)	57.6 (20.3)	47.3 (30.1)	54.4 (20.2)
Male	67.7 (21)	64.8 (16)	69.9 (21.7)	67.7 (17.3)	68.9 (21.3)	66.3 (16.7)
Age groups (years)						
≤ 25	38.4 (33.6)	47.7 (23.1)	45.7 (33.8)	57.3 (22.5)	41.6 (33.6)	51.9 (23.1)
26-35	53.1 (26.8)	54.3 (17.7)	62.1 (25.8)	60.3 (16.8)	57.2 (26.6)	57.1 (17.5)
36-45	61.9 (25.1)	58.9 (18.1)	56.6 (31.1)	62.1 (19.8)	59.1 (28.3)	60.5 (19)
≥46	48.8 (27.9)	58.4 (20.6)	51.1 (30.2)	61.9 (22.5)	50.1 (29.1)	60.4 (21.6)
Educational level						
Illiterate	49.8 (28.7)	51.8 (18.5)	56.7 (30.3)	59.4 (19.4)	53.3 (29.7)	55.7 (19.4)
Primary school	48.2 (32.6)	52.7 (22)	52.5 (33)	59.6 (22.4)	49.9 (32.5)	55.4 (22.2)
Religious education	61.8 (22.8)	67.9 (15)	51.7 (27.3)	66.8 (18.8)	56.1 (25.7)	67.3 (17.2)
College and above	70 (20.9)	72.5 (19.6)	87.5 (NA)	30 (NA)	72.9 (20)	65.4 (24.7)
Marital status						
Single	39.2 (39.4)	49.5 (29.1)	37.5 (37.5)	54.6 (22.6)	38.3 (37.5)	52.2 (25.3)
Married	52.5 (28.4)	54.7 (18.9)	57.4 (28.2)	61.5 (19.4)	54.9 (28.3)	58.1 (19.4)
Widowed	53.1 (13)	74.4 (12.3)	62.5 (54.5)	65.0 (30.4)	57.1 (33.2)	70.3 (20.2)
How long they have lived in the area						
< 10 years	52.9 (28.6)	53.3 (17.1)	53.5 (29.5)	59.8 (20.7)	53.2 (28.9)	56.5 (19.2)
> 10 years	51 (29.3)	56.2 (21.4)	56.7 (30.5)	61.4 (19.4)	53.8 (29.9)	58.8 (20.5)
Numbers of person per household						

≤ 5 person	53.2 (27.0)	51.9 (14.5)	61.5 (30.2)	57.3 (15.7)	56.7 (28.5)	54.1 (15.2)
6-8 person	50.9 (29.8)	55.8 (20.6)	52.6 (28)	59.9 (20.2)	51.7 (28.7)	57.9 (20.3)
> 8 person	51.0 (31.1)	58.7 (24.5)	52.8 (31.6)	64.6 (22.5)	52 (31.2)	61.9 (23.5)

Table S4. Knowledge and attitude scores of the participants by socio-demographic variables regarding antimicrobial resistance in Adadle district, Somali region, Ethiopia

Variables	Pastoralist		Agro-pastoralist		Overall	Overall
	Knowledge Mean (SD)	Attitude Mean (SD)	Knowledge Mean (SD)	Attitude Mean (SD)	Knowledge Mean (SD)	Attitude Mean (SD)
Sex						
Female	59.2 (8.1)	54.9 (10.3)	61.5 (6.9)	55.7 (7.5)	60.3 (7.6)	55.2 (9.1)
Male	61 (7.5)	56.3 (9.2)	64.1 (7.5)	57.4 (9.3)	63 (7.6)	56.9 (9.2)
Age groups (years)						
≤ 25	57.7 (10.8)	54.6 (9.7)	61.5 (7)	56.5 (7.4)	59.4 (9.4)	55.5 (8.8)
26-35	60.5 (7.4)	55.3 (10.2)	64.5 (5.9)	57.6 (6)	62.4 (7)	56.3 (8.6)
36-45	61.1 (6.1)	56 (10.2)	61.5 (6.8)	55.9 (7.7)	61.3 (6.4)	55.9 (8.7)
≥46	58.4 (7.9)	55 (10.5)	60.9 (8.6)	54.5 (11.1)	59.8 (8.3)	54.7 (10.7)
Educational level						
Illiterate	59.8 (7.7)	54.5 (9.7)	62.7 (7.6)	55.9 (8)	59.4 (7.8)	55.2 (8.9)
Primary school	58.3 (8.5)	55.6 (11.1)	61.5 (7.2)	54.9 (8.3)	61.3 (8.1)	55.3 (9.1)
Religious education	60.1 (8.2)	58.4 (9.6)	61.8 (5.3)	57.9 (8.3)	61.1 (6.7)	58.1 (8.8)
College and above	67 (6.4)	55.7 (8.5)	61.9 (NA)	54.1 (NA)	66.1 (6.2)	55.4 (7.6)
Marital status						
Single	55.4 (11.2)	55.4 (9.4)	61.2 (6.4)	55.1 (7.6)	58.7 (9.1)	55.2 (8.3)
Married	60.1 (7.8)	55.3 (10.1)	62.5 (7.1)	56.4 (7.9)	61.3 (7.6)	55.8 (9.1)
Widowed	58.3 (3.2)	58.5 (7.3)	65.1 (7.2)	52.4 (17.1)	61.2 (5.9)	55.9 (11.6)
How long they have lived in the area						
< 10 years	60.1 (8.5)	54.3 (9.9)	61.8 (6.7)	55.5 (7.6)	60.9 (7.7)	54.8 (8.9)
> 10 years	60 (7.6)	56.1 (10)	62.7 (7.4)	56.7 (8.5)	61.1 (7.6)	56.4 (9.2)
Numbers of person per household						
≤ 5 person	60.5 (7.4)	55.5 (8.2)	65.2 (6.1)	57.7 (6.9)	62.4 (7.2)	56.4 (7.8)
6-8 person	59.3 (8.7)	54.3 (11.5)	61.5 (7.4)	56.1 (8.2)	60.4 (8.1)	55.2 (9.9)
> 8 person	59.4 (8.1)	56.2 (10.5)	60.7 (7.2)	55 (8.9)	60.1 (7.6)	55.5 (9.7)

Table S5. Predictors of knowledge towards climate change in Adadle district, Somali region, Ethiopia.

Variables	Event Rate	OR[1]	95% CI[1]	*p*-value
Age group				
< 25	30 / 63 (48%)	—	—	
26-35	75 / 124 (60%)	1.51	0.72, 3.19	0.3
36-45	71 / 102 (70%)	2.30	1.04, 5.11	0.040
> 46	41 / 73 (56%)	0.99	0.42, 2.35	>0.9
Educational status				
Illiterate	137 / 235 (58%)	—	—	
Primary school	34 / 58 (59%)	1.46	0.68, 3.14	0.3
Religious learning	42 / 63 (67%)	1.54	0.80, 2.96	0.2
College and above	4 / 6 (67%)	0.84	0.12, 6.05	0.9
Marital status				
Single	11 / 25 (44%)	—	—	
Married	201 / 327 (61%)	5.92	1.66, 21.1	0.006

Variables	Event Rate	OR[1]	95% CI[1]	*p*-value
Divorced	1 / 3 (33%)	1.03	0.04, 30.1	>0.9
Widowed	4 / 7 (57%)	3.45	0.44, 27.2	0.2
**Occupation**				
Housewife	113 / 212 (53%)	—	—	
Government employee	12 / 19 (63%)	6.45	1.48, 28.0	0.013
Pastoralist	44 / 56 (79%)	5.18	2.33, 11.5	<0.001
farmer	12 / 23 (52%)	0.73	0.28, 1.92	0.5
Business	36 / 52 (69%)	3.45	1.50, 7.92	0.003

[1]OR = Odds Ratio, CI = Confidence Interval

Table S6. Predictors of attitude towards climate change in Adadle district, Somali region, Ethiopia.

Variables	Event Rate	OR[1]	95% CI[1]	*p*-value
Sex				
Female	122 / 257 (47%)	—	—	
Male	72 / 105 (69%)	3.14	1.44, 6.88	0.004
Age				
< 25	27 / 63 (43%)	—	—	
26-35	64 / 124 (52%)	1.54	0.75, 3.16	0.2
36-45	60 / 102 (59%)	1.76	0.80, 3.85	0.2
> 46	43 / 73 (59%)	1.59	0.68, 3.70	0.3
Occupation				
Housewife	107 / 212 (50%)	—	—	
Government employee	10 / 19 (53%)	0.85	0.24, 2.99	0.8
Pastoralist	33 / 56 (59%)	0.62	0.25, 1.53	0.3
Farmer	11 / 23 (48%)	0.19	0.06, 0.62	0.006
Business owner	33 / 52 (63%)	0.81	0.36, 1.84	0.6
Educational status				
Illiterate	111 / 235 (47%)	—	—	
Primary school	30 / 58 (52%)	1.55	0.76, 3.17	0.2
Religious learning	50 / 63 (79%)	4.13	2.03, 8.41	<0.001
College and above	3 / 6 (50%)	1.21	0.19, 7.75	0.8
How long they have lived in the area				
< 10 years	73 / 153 (48%)	—	—	
> 10 years	121 / 209 (58%)	1.46	0.90, 2.38	0.13

[1]OR = Odds Ratio, CI = Confidence Interval

Table S7. Predictors of knowledge towards antimicrobial and antimicrobial resistance in Adadle district, Somali region, Ethiopia.

Variables	Event Rate	OR[1]	95% CI[1]	*p*-value
Sex				
Female	106 / 257 (41%)	—	—	
Male	66 / 105 (63%)	5.48	2.40, 12.5	<0.001
Educational status				
Illiterate	122 / 235 (52%)	—	—	
Primary school	22 / 58 (38%)	0.79	0.38, 1.64	0.5
Religious learning	23 / 63 (37%)	0.49	0.25, 0.95	0.035
College and above	5 / 6 (83%)	14.0	0.87, 225	0.062
Age				

Variables	Event Rate	OR[1]	95% CI[1]	*p*-value
< 25	19 / 63 (30%)	—	—	
26-35	71 / 124 (57%)	2.39	1.17, 4.89	0.017
36-45	52 / 102 (51%)	1.98	0.91, 4.31	0.086
> 46	30 / 73 (41%)	1.49	0.63, 3.50	0.4
Occupation				
Housewife	96 / 212 (45%)	—	—	
Government employee	6 / 19 (32%)	0.25	0.05, 1.19	0.081
Pastoralist	32 / 56 (57%)	0.40	0.16, 1.01	0.052
Farmer	8 / 23 (35%)	0.23	0.07, 0.73	0.013
Business owner	30 / 52 (58%)	1.07	0.48, 2.39	0.9
Numbers of person per household				
< 5	75 / 129 (58%)	—	—	
6–8	53 / 121 (44%)	0.56	0.32, 0.97	0.038
> 8	44 / 112 (39%)	0.51	0.28, 0.95	0.035

[1]OR = Odds Ratio, CI = Confidence Interval

Table S8. Predictors of attitude towards antimicrobial and antimicrobial resistance at multivariable level in Adadle district, Somali region, Ethiopia.

Variables	Event Rate	OR[1]	95% CI[1]	*p*-value
Sex				
Female	139 / 257 (54%)	—	—	
Male	65 / 105 (62%)	2.64	1.28, 5.47	0.009
Educational status				
Illiterate	125 / 235 (53%)	—	—	
Primary school	32 / 58 (55%)	1.17	0.59, 2.34	0.6
Religious learning	44 / 63 (70%)	1.99	1.06, 3.74	0.033
College and above	3 / 6 (50%)	1.17	0.20, 6.74	0.9
Age				
< 25	34 / 63 (54%)	—	—	
26-35	74 / 124 (60%)	1.10	0.56, 2.16	0.8
36–45	57 / 102 (56%)	0.90	0.44, 1.84	0.8
> 46	39 / 73 (53%)	0.86	0.39, 1.87	0.7
Occupation				
Housewife	126 / 212 (59%)	—	—	
Government employee	10 / 19 (53%)	0.48	0.14, 1.58	0.2
Pastoralist	30 / 56 (54%)	0.37	0.16, 0.87	0.022
Farmer	14 / 23 (61%)	0.48	0.16, 1.43	0.2
Business owner	24 / 52 (46%)	0.33	0.15, 0.71	0.005

[1]OR = Odds Ratio, CI = Confidence Interval

Table S9. the percentage of householders' knowledge, attitude, and practice (KAP) scores in Adadle district, Somali region, Ethiopia.

	Agro pastoralist (N=179)	Pastoralist (N=183)	*p*-value
Knowledge about Climate Change			
Mean (SD)	55.3 (30.1)	51.9 (28.9)	0.265
Median [Min, Max]	62.5 [0, 100]	56.3 [0, 100]	
Attitude about Climate Change			
Mean (SD)	60.7 (20.0)	55.0 (19.7)	0.00623

	**Agro pastoralist (N=179)**	**Pastoralist (N=183)**	***p*-value**
Median [Min, Max]	60.0 [20.0, 90.0]	55.0 [10.0, 100]	
Climate Change practice			
Mean (SD)	20.4 (24.1)	23.6 (25.2)	0.218
Median [Min, Max]	16.7 [0, 100]	33.3 [0, 100]	
Knowledge about AMR			
Mean (SD)	50.1 (20.5)	47.2 (15.2)	0.131
Median [Min, Max]	53.3 [6.67, 93.3]	46.7 [6.67, 100]	
Attitude about AMR			
Mean (SD)	56.2 (8.13)	55.3 (10.0)	0.354
Median [Min, Max]	57.6 [30.6, 75.3]	56.5 [29.4, 80.0]	
Antibiotic use and AMR practice			
Mean (SD)	40.1 (16.7)	42.8 (14.1)	0.103
Median [Min, Max]	42.9 [0, 71.4]	42.9 [14.3, 75.0]	
